# Japanese Consensus Document on NexoBrid^®^, a Burn Eschar Removal Agent

**DOI:** 10.3390/ebj7020029

**Published:** 2026-05-13

**Authors:** Hajime Matsumura, Takahiro Ueda, Rei Ogawa, Yasuhiko Kaita, Hiroyuki Sakurai, Kazutaka Soejima, Junichi Sasaki

**Affiliations:** 1 Department of Plastic and Reconstructive Surgery, Tokyo Medical University, 6-7-1 Nishishinjyuku, Shinjukuku, Tokyo 160-0023, Japan; 2 Advanced Emergency and Critical Care Medical Center, Tottori University Hospital, Yonago, Tottori 683-8504, Japan; 99-uechanman@tottori-u.ac.jp; 3 Department of Plastic, Reconstructive and Aesthetic Surgery, Nippon Medical School Hospital, Bunkyo-ku, Tokyo 113-8603, Japan; r.ogawa@nms.ac.jp; 4 Department of Trauma and Critical Care Medicine, Kyorin University School of Medicine, Mitaka, Tokyo 181-8611, Japan; 5 Department of Plastic and Reconstructive Surgery, Tokyo Women’s Medical University, Shinjuku-ku, Tokyo 161-8666, Japan; sakurai.hiroyuki@twmu.ac.jp; 6 Department of Plastic and Reconstructive Surgery, Nihon University School of Medicine, Itabashi-ku, Tokyo 173-8610, Japan; soejima.kazutaka@nihon-u.ac.jp; 7 Department of Emergency and Critical Care Medicine, Keio University School of Medicine, Shinjuku-ku, Tokyo 160-8582, Japan; sasaki.junichi@keio.jp

**Keywords:** burn eschar, early removal, enzymatic debridement, purified product of pineapple stem juice extract, statement

## Abstract

**Highlights:**

**What are the main findings?**

- A Japan-specific Delphi consensus on NexoBrid^®^ was developed by seven burn specialists.
- 27 statements were formulated, of which 21 achieved expert consensus.

**What are the implications of the main findings?**

- The consensus provides practical, Japan-adapted guidance for the safe and effective use of NexoBrid^®^.
- It helps clinicians make evidence-informed decisions when applying NexoBrid^®^ in diverse burn scenarios.

**Abstract:**

Background: NexoBrid^®^ (NXB), an enzymatic debridement agent approved in Japan in 2022, provides a less invasive alternative to surgical excision for burn treatment. However, its optimal therapeutic benefit depends on appropriate patient selection and proper application technique. Existing international consensus documents are not fully applicable to clinical practice in Japan because of differences in available devices and drugs. Therefore, a Japan-specific consensus document was developed by a panel of seven Japanese burn experts, including four plastic surgeons and three emergency physicians. Methods: A questionnaire-based survey was conducted using the Delphi method to achieve expert consensus. Consensus was defined as agreement by at least 80% of committee members for each statement. A total of 27 statements were evaluated over two rounds. Results: Consensus was achieved for 20 of 27 statements (74.1%) in the first round and for 21 of 27 statements (77.8%) in the second round. The finalised statements were organised into seven categories according to their attributes: indications, pain management, application timing, application technique, post-application wound care, skin grafting, and scarring/aesthetic outcomes. Conclusions: This consensus document integrates the opinions of plastic surgeons and emergency physicians in Japan, while also incorporating relevant international perspectives, to provide practical guidance on the use of NXB for burn treatment. It is intended to improve the quality of burn care by clarifying efficacy, safety, and precautions associated with NXB use. These recommendations should be updated as further clinical experience accumulates.

## 1. Introduction

Eschar from burns often becomes an environment conducive to bacterial proliferation and worsens prognosis in burn patients when infection or sepsis develops. Local inflammation further damages surrounding healthy tissues, and the progression of the burn wound deteriorates the patient’s overall condition, underscoring the importance of early eschar removal. The Japanese Clinical Practice Guidelines for the Management of Burn Care recommend early eschar removal for extensive burns in the early phase following injury (within one week) [1].

Surgical excision remains the standard of care for eschar removal. It reliably removes necrotic tissue; however, excessive debridement or excision may result in unnecessary removal of viable tissue [2]. It also causes substantial blood loss and is often difficult to perform early after injury when the patient’s condition is unstable. NexoBrid^®^ (NXB) can address these limitations and is widely used for early eschar removal in Japan and other countries. NXB is a topical enzyme formulation derived from pineapple stems that lyses and removes eschar after four hours of application. Given that it exhibits high enzymatic activity against gelatine generated by thermal denaturation of dermal collagen, NXB is considered to act selectively on eschar. Hence, it is particularly useful at anatomical sites where preservation of healthy tissue is critical for functional or aesthetic outcomes, such as the hands and face. Moreover, a clinical study reported less blood loss with NXB than with surgical excision [3]. To maximise the benefits of NXB, clinicians must understand appropriate case selection and correct use.

In countries where NXB was approved earlier, mainly in Europe, consensus documents have been released that outline candidate wounds and recommendations for wound care and skin grafting after NXB application [4,5,6,7,8]. Although it has demonstrated treatment efficacy since the start of clinical use in Japan, numerous cases have been recorded with inadequate responses or challenging wound care after NXB application. Despite the release of multiple overseas consensus documents, none are fully applicable to Japan because available dressings, medical devices and drugs differ between Japan and other countries. Therefore, here, we aimed to develop a Japan-specific consensus document in order to better guide burn care employing NXB-based treatment.

This article is an English translation of a consensus document originally published in Japanese in the *Japanese Journal of Burn Injuries*, Volume 52, Issue 1 (March 2026) [9], reproduced with permission from the copyright holder.

## 2. Materials and Methods

Consensus development was conducted using the Delphi method, which summarises expert opinions through multiple rounds of anonymous questionnaires [10]. The consensus committee consisted of seven members: four plastic surgeons and three emergency physicians selected based on the following criteria: extensive experience and expertise in burn treatment, clinical experience with NXB, and recognition as Japanese burn experts.

Statements were initially drafted based on previous studies and refined through multiple panel meetings. During these meetings, statements addressing issues and questions encountered in clinical practice in Japan were added.

Consensus formation occurred in the following sequence:

First round: Committee members answered the questionnaire without knowledge of other members’ responses.

Second round: Committee members completed a second questionnaire while reviewing anonymized first-round responses.

The questionnaire included the following five choices:

1. Strongly agree.
2. Agree.
3. Neither agree nor disagree.
4. Disagree.
5. Strongly disagree.

Agreement was defined as selecting “1” or “2”, while consensus was established when agreement among 80% or more committee members concurred, indicating that at least six of the seven members agreed. After the second round, a panel meeting was convened to discuss whether any statements required revocation or modification. Statements were finalised with the agreement of all committee members.

## 3. Results

A total of 27 statements were formulated and classified into seven categories: indication, pain management, application timing, application technique, wound care after application, skin grafting, and scarring (aesthetics).

In the first round, agreement was achieved for 20 of the 27 statements (74.1% agreement; disagreement: statements 4, 5, 6, 17, 18, 23, and 26). In the second round, agreement was achieved for 21 of the 27 statements (77.8% agreement; disagreement: statements 4, 5, 17, 18, 23, and 26; Table 1). After the second-round questionnaire, a panel meeting determined that no re-voting was required to modify five statements (Statements 1, 10, 13, 14, and 25), after which the statements and consensus were finalised.

### 3.1. Statements with Agreement Achieved

#### 3.1.1. NexoBrid^®^ Is Advisable for Application to Burn Wounds with Mixed Depth Burn Patterns (All Seven Consensus Committee Members Agreed (7/7))

As NXB selectively removes eschar, its use is desirable in mixed-depth burn patterns that consist primarily of deep dermal burns (DDB). Mixed-depth patterns may include superficial dermal burns (SDB), but NXB is indicated for use in DDB and deep burns (DB). Therefore, it should not be applied to burn wounds that consist only or predominantly of SDB. Committee members have encountered mixed post-marketing cases involving SDB and DDB, including cases where intradermal hematomas in SDB failed to resolve and subsequently progressed to necrosis, complicating management after NXB application.

#### 3.1.2. NexoBrid^®^ Is Advantageous When Applied to Burns in Critical Areas (Such as the Face, Hands, Feet, and Perineum) (7/7)

As NXB is a topical formulation, it can be easily applied to anatomically complex areas where surgical excision is technically difficult [11,12,13,14]. In addition, its eschar-selective debridement preserves dermal components, potentially contributing to favourable aesthetic outcomes and functional outcomes.

#### 3.1.3. Paediatric or Elderly Patients Are Good Candidates for NexoBrid^®^ (6/7)

Given that NXB is associated with less blood loss than surgical excision [3], it may be beneficial for elderly patients with unstable general conditions early after injury. It can also reduce the need for surgery (e.g., surgical debridement or skin grafting) or the extent of surgery [15], rendering it useful for elderly patients for whom minimally invasive treatment is preferred. However, caution is warranted in elderly patients, as delayed wound healing or recurrent necrosis may occur when peripheral circulation is compromised (e.g., by arteriosclerosis or diabetes) [16].

A review of clinical and controlled studies comparing the standard of care has shown that NXB reduces the need for surgery or the extent of surgery in paediatric patients [17,18], indicating its usefulness in paediatric burns, which carry a substantial psychological burden for patients and families. However, NXB in paediatric patients is associated with procedure-related pain, potentially rendering the maintenance of a resting state for four hours difficult in a patient; therefore, appropriate analgesic and sedative management is required.

#### 3.1.4. Chemical Burns and Electrical Burns Are Not Amenable to NexoBrid^®^ (6/7)

Although this statement did not achieve agreement in the first round (5/7), consensus was reached in the second round (6/7). No clinical studies have been conducted on the effects of NXB in chemical or electrical burns. Although debridement completion with NXB has been reported in three of four cases of chemical burns and both cases of electrical burns [19], and NXB may be beneficial in the presence of denaturation to gelatine, the number of reported cases was insufficient to support a recommendation. One committee member had experience with the product for a case of chemical burns (alkali) but observed an inadequate response.

#### 3.1.5. NexoBrid^®^ Shows Inadequate Efficacy in Burns Caused by Low Temperatures (Less than 70 °C) (6/7)

NXB acts on gelatine generated by the thermal denaturation of dermal collagen. In burns caused by low temperatures, gelatinization is incomplete, reducing NXB efficacy. Thus, the use of NXB requires confirmation of the mechanism and type of injury.

#### 3.1.6. NexoBrid^®^ Is Less Likely to Be Efficacious in Third-Degree Burns Involving Damage Extending into the Subcutaneous Tissue and in Areas of Dry Black Eschar (6/7)

As NXB exerts its effects via gelatinase activity, it is less likely to act on adipose tissue. Therefore, it may be less efficacious in third-degree burns with thermal injury extending into the subcutaneous tissue. In addition, in dry necrotic tissue, such as black eschar, drug penetration throughout the necrotic tissue is less likely, reducing efficacy. If NXB is to be applied in such cases, the eschar should be adequately macerated by overnight pre-soaking (from the day before to immediately before application). Additional useful strategies include making an incision in the eschar before application of NXB to facilitate penetration or performing surgical excision of (only the superficial eschar layer to the extent that bleeding does not occur.

#### 3.1.7. Pretreatment Before NexoBrid^®^ with Topical Agents or Dressings Containing Silver or Iodine Should Be Avoided (7/7)

Since silver or iodine inhibits the enzymatic activity of NXB, topical agents or dressings containing these substances should not be used before NXB application [20]. Moreover, occlusive dressings containing silver or iodine should not be used during the 4 h period while NXB is in place. As the previous physician may have used topical agents or dressings containing silver or iodine, prior use should be verified before applying NXB. Notably, topical agents or dressings containing silver or iodine may be used for wound care after completion of the post-soaking following NXB application.

#### 3.1.8. Any Blister Roofs or Epidermal Keratin Should Be Removed Before the Application of NexoBrid^®^ (7/7)

Considering that NXB-mediated eschar removal requires direct enzymatic contact with the eschar, any physical barriers, such as blister roofs (epidermal keratin layers), must be removed. If blister roofs are present, they may indicate SDB, in which case NXB should not be applied.

#### 3.1.9. NexoBrid^®^ Should Not Be Applied Directly to Surgical Escharotomy Wounds and Macerated Wounds (7/7)

In a single-dose and repeat-dose intravenous toxicity study in pigs, changes in blood coagulation parameters and bleeding tendency were observed [21]. In clinical settings, a post-marketing survey in Japan reported five bleeding-related adverse drug reactions in five patients, with three events in three patients classified as serious. Of the serious events, two in two patients may have occurred as a result of NXB contacting relaxed incision wounds [22]. To prevent bleeding, NXB should not be applied to surgical escharotomy or macerated wounds. If NXB is applied in such cases, the wounds should be protected with Vaseline ointment or gauze coated with Vaseline ointment.

#### 3.1.10. Appropriate Pain Management Is Needed at the Application and Removal of NexoBrid^®^ (7/7)

As pain occurs during the application and removal of this drug, pain management should be initiated before both procedures. In particular, as body movements may impair the efficacy of NXB or cause adverse events in children, adequate sedative and analgesic management is required.

#### 3.1.11. Local Anaesthesia or Nerve Blocking Is Useful in Cases of Injuries Limited to the Extremities (7/7)

In cases of injuries limited to the extremities, this drug does not routinely need to be used in an operating room but can be applied at the bedside for pain management with local anaesthesia or nerve blocking. Overseas consensus documents note that burns of the extremities are manageable under local anaesthesia [5,8].

#### 3.1.12. NexoBrid^®^ Is More Likely to Be Beneficial When Applied in the Early Phase Following Injury (7/7)

NXB can be more beneficial when applied before necrotic tissues become dry. Clinical studies have demonstrated that application within 84 h of injury is associated with high efficacy in eschar removal [3,15,23]. In addition, early eschar removal is expected to reduce the risk of infection. However, burns caused by high-temperature liquids may involve inadequately denatured dermal collagen; in such cases, the drug should be applied beyond 72 h after injury [5].

#### 3.1.13. Burn Wounds That Are Immediately After Injury and Still Adequately Moist Do Not Require Pre-Soaking (7/7)

The European consensus document states that very early (within 12 h after injury) cases in which necrotic tissues remain adequately moist do not require pre-soaking [5]. One committee member encountered a case in which an early application of NXB, without pre-soaking, achieved adequate eschar removal. Given that extensive experience with NXB is required to determine when pre-soaking can be skipped, early use of the drug should follow the standard procedure. In contaminated burn wounds, adequate cleansing is required regardless of whether pre-soaking is omitted.

#### 3.1.14. Burn Wounds in Which Necrotic Tissues Have Hardened over Time After Injury Need to Be Adequately Macerated with Prolonged Pre-Soaking (7/7)

Given that eschar removal by NXB depends on enzymatic activity, the onset of action requires penetration into the eschar. Thus, the onset of action is less likely in dry, hardened necrotic tissues due to insufficient penetration. If NXB is to be applied to such hard necrotic tissues, they should be adequately macerated by pre-soaking beginning on the day before application [4]. There was agreement that pre-soaking could be initiated the day before application, allowing the procedure to be completed during the daytime shift.

#### 3.1.15. Pseudo-Eschar Is a Surface Debris to the Wound Surface That Originates from Exudate Fluid or Drug Residues After Removal of NexoBrid^®^ (7/7)

Pseudo-eschar is a surface debris to the wound surface formed after application of NXB and is considered different from necrotic tissues, resulting from necrosis of the zone of stasis [4]. It is formed on a relatively shallow wound surface and may be formed as early as immediately after post-soaking.

#### 3.1.16. To Determine Eschar Removal and Differentiate from Pseudo-Eschar Formation, Images Should Be Captured at Three Time Points: Before Application of NexoBrid^®^, After Application of NexoBrid, and Post-Soaking (7/7)

Determining eschar removal and distinguishing pseudo-eschar from true eschar requires further investigation. Notably, wound conditions should be monitored and assessed over time. Images should be captured and compared before the application of NXB, after the application of NXB (immediately after removal), and immediately after soaking. If petechial haemorrhaging is observed immediately after NXB removal but a white surface debris is noted after post-soaking, the surface debris may be a pseudo-eschar.

#### 3.1.17. Aggressive Removal of Pseudo-Eschar Is Not Necessary (6/7)

Pseudo-eschar is part of the healing process and does not require surgical intervention as it spontaneously desquamates or resolves [4]. Some consider pseudo-eschar to function as a biological dressing. However, if early wound closure is desired, removal of the pseudo-eschar followed by skin grafting may be appropriate. This statement indicates that preservative treatment is feasible but does not recommend excision or skin grafting.

#### 3.1.18. After Soaking, the Wound Surface Should Be Maintained in a Moist Environment (7/7)

Considering that post-soaking dryness may contribute to the progression of necrosis or pseudo-eschar formation, maintaining a moist environment, which can facilitate healing and reduce the frequency of dressing replacement and the pain associated with replacement [7]. Acceptable dressing materials include those routinely used to maintain moisture in burn treatment, such as Vaseline gauze, non-adherent silicone gauze, and hydrocolloids.

#### 3.1.19. Trafermin Is Effective Even for Wounds After NexoBrid^®^ Removal (7/7)

Trafermin (FIBLAST^®^ Spray: Kaken Pharmaceutical Co., Ltd., Tokyo, Japan) is a formulation of recombinant human basic fibroblast growth factor indicated for the treatment of burn ulceration. NXB is a proteolytic enzyme shown to achieve eschar removal in a mouse burn model and does not impair burn wound healing, even when trafermin is applied to wounds after NXB treatment [24].

#### 3.1.20. Skin Grafting Should Be Performed Immediately if Spontaneous Epithelialization Is Less Likely to Occur After NexoBrid^®^ Removal (7/7)

Although overseas consensus documents suggest considering skin grafting in DDB cases expected to heal conservatively, epithelialization may not occur even 21 days after NXB removal [5,7,8], and some panellists support earlier grafting without waiting for 21 days. If spontaneous epithelialization is unlikely, skin grafting should be performed immediately. Given that burns caused by high-temperature liquids may deepen after NXB application, wounds should be carefully observed after removal, and the timing of grafting should be determined accordingly. Standard skin grafting procedures used in routine burn treatment are acceptable. In other countries, cryopreserved allogeneic skin grafting has been performed after NXB application and is recommended for managing wound surfaces that are likely or unlikely to epithelialize after post-soaking [5]. In Japan, cryopreserved allogeneic skin grafting is managed by the Japan Skin Bank Network, and measures to relax shipping criteria have been implemented in severe cases where NXB is used for lifesaving purposes, although these measures are provisional [25]. However, such cases of cryopreserved allogeneic skin grafting after NXB application remain limited in Japan and require additional experience.

#### 3.1.21. NexoBrid^®^ Provides an Aesthetically More Favourable Outcome than Surgical Excision (6/7)

NXB can preserve healthy tissues through eschar-selective debridement and is therefore expected to provide aesthetically favourable outcomes. At a one-year follow-up, an overseas clinical study reported that the modified Vancouver Scar Scale (mVSS) was significantly more favourable in the NXB group than in the standard-of-care group [3]. In contrast, a meta-analysis found that mVSS was more favourable in the NXB group than in the standard care group, although this difference did not reach statistical significance [26]. Further investigation is warranted. Some panellists noted that debridement and subsequent wound management influence aesthetic outcomes and that aesthetics may be difficult to assess at the debridement stage alone.

### 3.2. Statements with No Agreement

#### 3.2.1. The Use of NexoBrid^®^ in Circumferential Burns of the Extremities in the Early Phase Following Injury Can Avoid Surgical Escharotomy (3/7)

As some panellists stated that avoiding surgical escharotomy with NXB is possible but not certain, consensus was not achieved. Some panellists expressed that appropriate use by physicians with extensive case experience might avoid the need for surgical escharotomy. One of the panellists has encountered a case in which the use of NXB reduced the compartment pressure in circumferential burns of the hands and forearms. Notably, a surgical escharotomy should be immediately made in cases of established burn-induced compartment syndrome (BICS).

#### 3.2.2. The Use of NexoBrid^®^ Can Complete Debridement of the Whole Burn Wound in the Early Phase Following Injury (5/7)

NXB can be applied in the early phase after injury, when the patient’s general condition is unstable, thereby enabling earlier completion of debridement. However, some panellists noted that additional surgical excision may be required or that recurrent necrosis may occur in some cases, thereby precluding agreement on this statement. To complete early debridement after injury, appropriate case selection, adequate preparation, and accurate assessment of wounds after NXB application are essential. In cases with extensive burns, combining surgical excision with NXB may achieve complete debridement earlier than surgical excision alone [27].

#### 3.2.3. Burn Depth Can Be Determined by the Colour or Bleeding Pattern After Application of NexoBrid^®^ (4/7)

This statement did not reach agreement because some panellists noted that accurate assessment requires experience and cannot be reliably performed by all clinicians, although colour or bleeding patterns may assist in determining burn depth [28,29].

#### 3.2.4. Antiseptic Solutions Should Be Used After Soaking Whenever Possible (2/7)

Although antiseptic solutions are not essential for post-soaking, their use is recommended when necessary. In an overseas Phase II study, fever and wound infections occurred more frequently in the NXB group than in the standard-of-care group. In a subsequent overseas Phase III study, maceration with antiseptic solution-impregnated gauze was used as a preventive measure, reducing the risk of fever and wound infection [30]. A 0.05% chlorhexidine solution is commonly used as an antiseptic solution, and some overseas institutions use a Prontosan solution [31].

#### 3.2.5. A Reddish Black Wound Surface After Removal of NexoBrid^®^ Heals Spontaneously with Conservative Treatment (1/7)

A reddish-black wound surface noted after application of NXB is typically considered the SDB site. This haematoma results from the disruption of blood vessels in the superficial dermal layer and may resolve with conservative treatment, as it is absorbed and ultimately resolves. However, some panellists reported cases without spontaneous healing, preventing consensus. Panellists expressed the opinion that a wound surface often turned reddish black after application of NXB in cases of poor microcirculation, such as elderly patients or patients with diabetes.

#### 3.2.6. Skin Grafting Is Also Feasible on the Day of Application of NexoBrid^®^ (3/7)

Given that determining eschar removal requires experience and that the risk of graft dislodgement due to residual eschar is substantial, some panellists emphasised the need for careful assessment; consensus was not reached. However, this statement is not a recommendation against skin grafting on the day of NXB application. The European consensus document recommends that skin grafting should be performed at least two days after NXB application [5]. Some panellists reported that skin grafting may be feasible on the day of application if eschar removal is adequate and the drug is thoroughly removed with cleansing. Although no panellists had personal experience performing grafting on the day of NXB application, some reported favourable engraftment with grafting on the following day. In Japan, one report described favourable outcomes when skin grafting was performed on the day of superficial-layer removal following NXB application and hydrosurgical debridement [32].

## 4. Discussion

NXB, a burn eschar removal agent, is a useful alternative to surgical excision based on its eschar-selective action, reduced blood loss, shorter time to completion of eschar removal, and other advantages [3,15]. This consensus document summarises both Japanese and overseas evidence, as well as the opinions of panellists with extensive experience and expertise in burn treatment, using the Delphi method. Consequently, 27 statements were developed, of which 21 achieved agreement (77.8% agreement rate). The agreement rate tended to be lower than that reported in overseas consensus documents (European 2020: 97.7%, Spain: 97.2%) [5,8]. The following factors may have influenced: first, this document adopted a policy of avoiding ambiguous terms such as “potentially” or “expected” whenever possible; second, the proportion of panellists selecting “Neither agree nor disagree” was high, likely due to less clinical experience than in other countries, because statements were formulated 1.5 years after the start of clinical use.

Statements aligned in content with overseas consensus documents generally provided similar recommendations. However, Statements 4 (avoidance of surgical escharotomy), 17 (determination of burn depth based on colour and bleeding patterns), and 18 (antiseptic solutions for post-soaking) showed different results.

Statement 4 (Avoidance of surgical escharotomy) was formulated under the policy of avoiding ambiguous expressions such as “potentially” or “expected.” Thus, wording indicating that surgical escharotomy “can be avoided” was used. In addition, some panellists suggested avoiding assertive recommendations because close observation is required during the NXB application; therefore, consensus was not achieved. In contrast, several reports have described successful avoidance of BICS when NXB was applied as early as possible [12,33,34]. Krieger et al. [12] reported that NXB reduced compartment pressure from 35 to 75 mmHg to 25 mmHg without performing surgical escharotomy in four patients (seven hands). Fischer et al. [33] reported that NXB applied 2–22 h after injury avoided surgical escharotomy in 13 patients (20 upper limbs) with deep upper-limb burns, and Mataro et al. [34] reported reductions in compartment pressure to ≤30 mmHg within 1 h of injury in 23 patients with circumferential extremity burns. The European consensus document also notes the potential to avoid BICS [5]. One panellist reported a case in which NXB reduced compartment pressure in circumferential burns of the hands and forearms [35]. Continuous monitoring of compartment pressure before, during, and after NXB application, involving procedures such as the subcutaneous insertion of an injection needle and the connection of the needle to a probe for blood pressure, ensures safety. Although agreement was not reached, early use of NXB after injury under appropriate management by physicians with extensive clinical experience may reduce invasiveness in circumferential burns of the extremities.

For Statement 17 (Determination of burn depth by colour and bleeding patterns), some panellists stated that burn depth could be predicted from the colour or bleeding pattern of wounds after NXB application, but that accurate assessment requires experience; therefore, consensus was not achieved. Reports differ regarding the interpretation of a homogeneous reddish-black wound surface after NXB application: one suggests that it may allow spontaneous epithelialization because much of the dermis remains (DDB close to SDB) [28], whereas another indicates that it may represent DDB requiring prolonged epithelialization or, in some cases, skin grafting [29]. The discrepancy between these reports suggests that wound evaluation after NXB application can be difficult.

For Statement 18 (Antiseptic solutions for post-soaking), a substantial proportion of panellists chose “Neither agree nor disagree” due to concerns regarding the cytotoxic potential of antiseptic solutions; therefore, agreement was not achieved. However, antiseptic solutions are recommended when necessary. In an overseas Phase II study that did not recommend post-soaking with antiseptic solutions, the incidences of fever and wound infection were higher in the NXB group than in the standard-of-care group. In a subsequent overseas Phase III study, post-soaking with antiseptic solutions reduced the risks of fever and wound infection [30]. Accordingly, Japan’s risk management plan (RMP) identified fever and wound infection as important risks. Overseas package inserts state that antiseptic solution soaking should be performed [36], whereas the Japanese package insert notes that antiseptic solutions may be used as needed but does not mandate their use [24]. Nevertheless, the use of antiseptic solutions is advisable to reduce the risks of fever and wound infection as described above. One major cause of fever after application of the NXB occlusive dressing for 4 h is the insulating properties of the dressing that reduce normal heat dissipation. In surgery, using an occlusive dressing (a simple autoclaved polyurethane garbage bag) can keep a patient warm in cold operating rooms but often leads to excessive warming after a couple of hours, necessitating opening the occlusive dressing to dissipate the heat. One purpose of post-NXB soaking is to facilitate heat dissipation. Although concerns exist regarding the cytotoxic potential of antiseptic solutions, an in vitro study reported that chlorhexidine reduces cell viability in a concentration-dependent manner; however, the chlorhexidine concentration used for post-soaking (0.05%) is low and is therefore considered to have limited effects after two hours of soaking [37].

NXB is a topical formulation that is easy to use but requires appropriate wound care before application to maximise its therapeutic efficacy. The eschar removal by NXB is based on an enzymatic reaction and is inhibited by topical agents or dressing materials containing silver or iodine, which should not be used for wound care before application. Occlusive dressing used during the four-hour period in which the drug is kept in place should also not contain silver or iodine. Although it is acceptable to use topical agents or dressing materials containing silver or iodine after the post-soaking, following application of NXB, caution should be exercised because the use of topical agents or dressing materials containing silver is likely to cause the formation of pseudo-eschar tissue. In addition, because an adequate response to NXB itself needs to contact with necrotic tissue, hard necrotic tissue should be adequately moistened or macerated before NXB application. The enzymatic activity of NXB, consistent with the behaviour of all other enzymes, occurs within tissue that possesses sufficient moisture content. The eschar, which is the substrate for this enzymatic activity, should therefore remain sufficiently moist. Although package inserts explain that pre-soaking to reduce the bacterial load and moisten the eschar should be performed for approximately two hours, many institutions perform overnight pre-soaking in cases where some time has passed since injury. Skipping pre-soaking is acceptable for adequately moist and clean necrotic tissues when using NXB in the very early period after burn injury [5]. In addition, it should be noted that an appropriate volume relative to the area of application (one 5 g kit per 450 cm^2^) should be used.

Since the use of NXB provides eschar-selective debridement, decreases in the percentage of cases undergoing skin grafting and the area of skin grafting have been reported [15]. Although NXB is more likely to preserve the dermis necessary for a normal epithelialization process than conventional surgical excision, it should be used with the understanding that spontaneous epithelialization is not possible in all cases. The need for skin grafting has to be considered after carefully observing the wound surface before and after the application of NXB. For the timing of skin grafting, overseas consensus documents recommend considering skin grafting at 21 days or later in cases of delayed epithelialization despite preserved dermis [5], whereas this consensus document includes opinions supporting skin grafting without waiting for 21 days. Skin grafting should, in particular, be performed early at sites that are likely to present with scar contracture. Caution should be exercised because the development of pseudo-eschar, especially in dermal wounds (SDB and DDB), may interfere with graft take. In addition, for DB, the European consensus document recommends skin grafting at least two days after the application of NXB, whereas the Spanish consensus document recommends grafting at three to five days after the application of NXB [5,8]. This consensus document states that skin grafting may be performed less than two days after the application of NXB only in wounds in which complete eschar removal is considered to have been achieved with no drug residue. Deeper wounds usually have less pseudo-eschar formation. In Japan, a report described skin grafting performed on the day of the application of NXB [32], but the evidence is considered insufficient and did not lead to a recommendation (Statement 26). It should be noted that one panellist encountered a case in which skin grafting was performed on the day after the application of NXB with favourable outcomes. However, in some cases, another reason for delaying grafting by a day or more after NXB is practical from the perspective of workload distribution. More cases will be required to determine the optimal timing of grafting.

NXB must be used with an understanding of its characteristics and, in selected cases, to leverage its strengths. More specifically, such cases include burns in critical areas (face, hands, feet, and the perineal region) where surgical excision is technically difficult. Given that these areas are aesthetically and functionally important, NXB, which can selectively remove eschar, is useful. In addition, children and elderly individuals with very thin skin, which challenges the preservation of the non-injured dermis and for whom surgical stress should be avoided, are also good candidates. For elderly people, wound healing may be delayed, or recurrent necrosis may occur after the application of NXB in cases of poor peripheral circulation (such as arteriosclerosis and diabetes [16]), and further study is warranted.

Thermal denaturation of collagen has been reported to occur rapidly at 65 °C [38]. In addition, a study investigating the efficacy of NXB in eschar removal in human tissue (in vitro) simulating burns at 40 °C to 100 °C reported evidence of collagen denaturation at ≥60 °C and efficacy of NXB at ≥70 °C, with no evidence of efficacy at ≤50 [39]. In clinical settings, a significantly higher proportion of burns caused by high-temperature liquids required the addition of surgical excision than burns caused by flame [40]. However, several controlled studies, including burns caused by various mechanisms of injury, reported favourable efficacy [41,42], and application may be acceptable in burns caused by heat at ≥70 °C.

Cases not suitable for NXB include dry and hard necrotic tissue, where the drug is less likely to penetrate, and cases previously treated with topical agents or dressings containing silver or iodine. There is not sufficient evidence to make recommendations for its use in chemical burns and electrical burns. In particular, with chemical burns, it is difficult to determine whether gelatinization has occurred, and careful application is necessary. NXB is not suitable for low-temperature burns because gelatinization is unlikely to have occurred, which may result in inadequate efficacy.

This consensus document was developed to reflect clinical experience in Japan by referencing overseas consensus documents. While not included in overseas consensus documents, three original statements on pseudo-eschar tissue were formulated in Japan (Statements 19, 20, and 21). Pseudo-eschar is an important consideration in making full use of NXB. This consensus document will contribute to improving the quality of burn treatment with NXB because it describes methods for differentiating and managing pseudo-eschar. Recommendations included in this consensus document are not invariable and have to be updated as clinical experience accumulates or new evidence becomes available. The proper use of NXB for chemical burns, electrical burns, and scald burns warrants further investigation. In this consensus document, the formulation of statements and the formation of agreement occurred based on evidence from clinical studies, but the results also incorporated the opinions of panellists.

## 5. Conclusions

This consensus document integrates the opinions of Japanese plastic surgeons and emergency physicians with extensive experience in treating burns, along with evidence collected in Japan and overseas, to provide recommendations on the efficacy, safety, and precautions of NXB in burn treatment. These recommendations will help improve the quality of care for burn treatments with NXB in Japan.

## Figures and Tables

**Table 1:** Japanese consensus statements and results of voting as obtained via the Delphi method.

No.	Category	Statement	Result	Agreement
1	Indications	NexoBrid^®^ is advisable for application to burn wounds with mixed depth burn patterns.	Strongly agree: 1 Agree: 6	◯
2	Indications	NexoBrid^®^ is very beneficial when applied to burns in critical areas (face, hands and feet, perineum, etc.).	Strongly agree: 4 Agree: 3	◯
3	Indications	Paediatric or elderly patients are good candidates for NexoBrid^®^.	Strongly agree: 1 Agree: 5 Neither agree nor disagree: 1	◯
4	Indications	The use of NexoBrid^®^ in circumferential burns of the extremities in the early phase following injury can avoid surgical escharotomy.	Agree: 3 Neither agree nor disagree: 4	×
5	Indications	The use of NexoBrid^®^ can complete debridement of the whole burn wound in the early phase following injury.	Strongly agree: 2 Agree: 3 Neither agree nor disagree: 2	×
6	Indications	Chemical burns and electrical burns are not amenable to NexoBrid^®^.	Strongly agree: 4 Agree: 2 Neither agree nor disagree: 1	◯
7	Indications	NexoBrid^®^ shows inadequate efficacy in burns caused by low temperatures (less than 70 °C).	Strongly agree: 2 Agree: 4 Neither agree nor disagree: 1	◯
8	Indications	NexoBrid^®^ is less likely to be efficacious in third-degree burns involving damage extending into the subcutaneous tissue and in areas of dry black eschar.	Strongly agree: 2 Agree: 4 Neither agree nor disagree: 1	◯
9	Indications	Pretreatment before NexoBrid^®^ with topical agents or dressings containing silver or iodine should be avoided.	Strongly agree: 6 Agree: 1	◯
10	Indications	Any blister roofs or epidermal keratin should be removed before the application of NexoBrid^®^.	Strongly agree: 6 Agree: 1	◯
11	Indications	NexoBrid^®^ should not be applied directly on surgical escharotomy wounds and macerated wounds.	Strongly agree: 5 Agree: 2	◯
12	Pain management	Appropriate pain management is needed at the application and removal of NexoBrid^®^.	Strongly agree: 7	◯
13	Pain management	Local anesthesia or nerve blocking is useful in cases of injuries limited to the extremities.	Strongly agree: 3 Agree: 4	◯
14	Application timing	NexoBrid^®^ is more likely to be beneficial when applied in the early phase following injury.	Strongly agree: 3 Agree: 4	◯
15	Application timing	Burn wounds that are immediately after injury and still adequately moist do not require pre-soaking.	Strongly agree: 2 Agree: 5	◯
16	Application timing	Burn wounds in which necrotic tissues have hardened over time after injury need to be adequately macerated with prolonged presoaking.	Strongly agree: 3 Agree: 4	◯
17	Application Technique	Burn depth can be determined by the color or bleeding pattern after application of NexoBrid^®^.	Strongly agree: 3 Agree: 1 Neither agree nor disagree: 3	×
18	Wound care after application	Antiseptic solutions should be used after soaking whenever possible.	Strongly agree: 1 Agree: 1 Neither agree nor disagree: 5	×
19	Wound care after application	Pseudo-eschar is a surface debris to the wound surface that originates from exudate fluid or drug residues after removal of NexoBrid^®^.	Strongly agree: 4 Agree: 3	◯
20	Wound care after application	To determine eschar removal and differentiate from pseudo-eschar formation, images should be captured at three time points: before application of NexoBrid^®^, after application of NexoBrid^®^, and after post-soaking.	Strongly agree: 6 Agree: 1	◯
21	Wound care after application	Aggressive removal of pseudo-eschar is not necessary.	Strongly agree: 1 Agree: 5 Disagree: 1	◯
22	Wound care after application	After post-soaking, the wound surface should be a moist environment.	Strongly agree: 5 Agree: 2	◯
23	Wound care after application	A reddish black wound surface after removal of NexoBrid^®^ heals spontaneously with conservative treatment.	Agree: 1 Neither agree nor disagree: 5 Disagree: 1	×
24	Wound care after application	Trafermin is effective even in wounds after NexoBrid^®^ removal.	Strongly agree: 2 Agree: 5	◯
25	Skin grafting	Skin grafting should be performed immediately if spontaneous epithelialization is less likely to occur after NexoBrid^®^ removal.	Strongly agree: 7	◯
26	Skin grafting	Skin grafting is also feasible on the day of application of NexoBrid^®^.	Strongly agree: 1 Agree: 2 Neither agree nor disagree: 4	×
27	Scarring (esthetics)	NexoBrid^®^ provides an aesthetically more favorable outcome than surgical excision.	Strongly agree: 2 Agree: 4 Neither agree nor disagree: 1	◯

○ indicates agreement; × indicates disagreement.

## Data Availability

No new data were created or analysed in this study. Data sharing is not applicable to this article.

## Abbreviations

The following abbreviations are used in this manuscript:

NXB	NexoBrid
DDB	deep dermal burn
SDB	superficial dermal burn
DB	deep burn
mVSS	modified Vancouver Scar Scale
BICS	burn-induced compartment syndrome
RMP	risk management plan
